# Incorporating Family Function into Chronic Pain Disability: The Role of Catastrophizing

**DOI:** 10.1155/2016/6838596

**Published:** 2016-03-29

**Authors:** Fatemeh Akbari, Mohsen Dehghani, Ali Khatibi, Tine Vervoort

**Affiliations:** ^1^Family Research Institute, Shahid Beheshti University, G.C., Tehran 1983963113, Iran; ^2^Department of Psychology, Shahid Beheshti University, G.C., Tehran 1983963113, Iran; ^3^Psychology Department, Bilkent University, 06800 Ankara, Turkey; ^4^Interdisciplinary Program in Neuroscience, Bilkent University, 06800 Ankara, Turkey; ^5^Department of Experimental-Clinical and Health Psychology, Ghent University, 9000 Ghent, Belgium

## Abstract

*Background*. Observers' responses to pain are recently investigated to more comprehensively explain chronic pain (CP) and disability. However, the role of family context, defined as interference in roles, communication, and problem-solving, and how (i.e., through which mechanisms) these variables contribute to CP related disability have yet to be examined.* Objectives*. The aim of the present study is to examine family context in relationship to pain catastrophizing, fear of movement, and depression and its role in understanding CP disability. Three different models were examined.* Methods*. A total sample of 142 patients with musculoskeletal chronic pain was recruited to examine the role of fear of movement, pain intensity, pain catastrophizing, and depression in relationship to family functioning as predictors of disability.* Results*. Findings indicated that two models showed acceptable fit, but one of them revealed superior fit indices. Results of the model with superior fit indices indicated that family dysfunction may contribute to catastrophic thinking, which, in turn, contributes to patients' disability through increasing fear of movement and depression.* Discussion*. The current study provides further support for the notion that the impact of emotional and cognitive variables upon CP-related disability can be better understood when we consider the social context of pain patients and family function in particular.

## 1. Introduction

Contemporary conceptualizations of chronic pain (CP) suggest that cognitive and emotional variables contribute to disability in CP patients. The fear-avoidance model has been most influential in this regard and posits that pain catastrophizing is a potential precursor of pain-related fear which may cause patients to avoid activities. Avoidance behaviors may persist since they occur in anticipation of pain rather than as a response to pain [1]. While avoidance may have protective functions, this may be no longer the case in the context of CP: continued avoidance may interfere with pursuit of valued goals, thereby contributing to increased interference in daily functioning, and, in all likelihood, affect mood. Indeed, evidence suggests that, in the long term, persistent avoidance contributes to increased disability and functional impairment, as well as increased depressive symptoms [1, 2]. Thus, the fear-avoidance model of pain offers insight into one particular pathway through which cognitions (i.e., pain catastrophizing) may lead to maladaptive outcomes in chronic pain patients [1]. However, restricting the study of pain to the examination of intrapersonal psychological variables is insufficient to fully understand pain and associated outcomes such as disability and depressive symptoms. Empirical inquiry suggests that knowledge of other factors contributing to disability, especially observer responses to pain and more specifically family dynamics, is key in understanding pain-related problems. Indeed, according to biopsychosocial models of CP, disability is the result of multiple influencing variables [3, 4]. Recent studies are paying more attention to the social context of pain in order to further understand and explain the mediating and moderating factors between cognitions, emotions, and disability.

One mechanism by which pain-related cognitions such as pain catastrophizing may impact pain and disability is via the social environment in which the patient lives. For instance, recent studies conceptualize pain catastrophizing as a coping strategy with important interpersonal correlates (i.e., increased pain expression and increased responsiveness of others; see, e.g., [5–7]). Further, findings have shown that patients' long-term catastrophizing is likely to adversely impact family atmosphere, mainly by triggering negative responses from others, such as invalidating responses and unsupportive and rejective reactions [8, 9], which have, in turn, been found to contribute to worse outcomes such as depression and disability [9, 10]. Thus, patients' pain catastrophizing might adversely affect family functioning and impact the way that patient communicates with family members, doing his/her works (roles), and how they deal with possible conflicts and solve the problems. This situation can develop a context that may, in turn, contribute to increased pain-related fear and disability level. Moreover, such problematic interactions might also increase patient's depression [11], further contributing to increased disability.

Alternatively, chronic pain may also lead to increased activity limitations and changes in roles thereby imposing strain on one's family atmosphere [12, 13]. Such changes may impose additional burden on relationships among family members [14] and contribute to increasing levels of family distress. Besides, family's ability to solve daily problems may deteriorate as a result of extra focus on patient's pain and subsequent relational problems. Such a stressful family interaction may contribute to the patient's disability and depression [11]. More importantly, family dysfunction may trigger patients' pain catastrophizing which is another pathway to more disability and negative outcomes through extra focus on the pain problem.

In sum, empirical inquiry increasingly attests to the role of both individual and interpersonal variables, such as family context, in understanding pain and associated disability [9, 15, 16]. However, specific relationships between individual variables and family context are yet to be conceptualized in a tested model. The objective of the present study is to examine the role of both individual and family-related variables (family functioning indexed by roles, communication, and problem-solving) in understanding chronic pain-related disability. This study hypothesizes that pain experience is not merely an individual problem but impacts the system which patient lives in (i.e., family) and is likely to impose changes specifically on roles, communication, and problem-solving of the family. In addition, we assumed that patients' failure to fulfill these functions is associated with catastrophizing, fear of movement, and depressive mood as pathways towards disability.

Three models are tested in order to determine which model best explains disability in CPPs. In the first model, we hypothesized that catastrophizing contributes to decreased family function, which, in turn, contributes to increased fear of movement and depression leading to increased disability. Two alternative models were tested. Specifically, within the first alternative model, we assumed that family function predicts catastrophizing while other paths stay unchanged. The third model was developed based on the notion that family dysfunction might be a function of depression and disability. That is, family dysfunction may be better understood as a consequence of depression and disability.

## 2. Methods

### 2.1. Participants

Participants in this study were recruited from Atieh Hospital and Rasa Pain Clinic, Tehran, Iran. The study was approved by Shahid Beheshti University Research Ethics Committee and the Mental Health Center of Atieh Hospital. To be included, patients had to be at least 19 years old and experience pain for at least three months. Patients were excluded if they had brain injury or major cognitive dysfunction based on their medical records. All participants in this study provided informed consent and voluntarily participated in the study. The data were gathered over the course of 6 months (i.e., January to July 2013). One hundred and forty-two eligible patients with chronic musculoskeletal pain and their spouses agreed to participate in this study.

### 2.2. Measures

Participants completed a battery of questionnaires assessing pain intensity (VAS), fear of movement, pain catastrophizing, disability, depression, and Family Assessment Device (FAD).  Table 1 reports the means, standard deviations, and Cronbach's alphas. All measures indicated satisfactory internal consistency, at or above 0.70, except for PCS-magnification (alpha = 0.66).

### 2.3. Visual Analogue Scale (VAS)

The VAS is a 10-centimeter ungraded horizontal line with two anchors from 0 indicating “the minimum intensity of pain” to 100 indicating “the maximum intensity of pain.” The CPPs were asked to indicate their mean pain intensity in the last week. This scale has consistently demonstrated adequate validity and sensitivity to change [17].

### 2.4. Tampa Scale of Kinesiophobia (TSK)

The TSK assesses the participants' self-reported fear of movement or (re)injury [18, 19]. The TSK consists of 17 items (e.g., I am afraid that I might injure myself if I exercise) and participants are requested to rate each item on a 4-point Likert-type scale (0 = extremely disagree, 3 = extremely agree). The total score is calculated after reverse-scoring of 4 items [4, 8, 12, 16]. Higher scores reflect greater fear of movement. The TSK has shown acceptable reliability and validity in previous studies [19]. In the present sample, Cronbach's alpha for TSK was 0.80. This measure has been translated into Persian and its psychometric properties are good [20, 21].

### 2.5. Pain Catastrophizing Scale (PCS)

The PCS is a 13-item self-report scale that measures 3 dimensions of catastrophizing about pain: rumination (4 items: e.g., I anxiously want the pain to go away), magnification (3 items: e.g., I become afraid that the pain may get worse), and helplessness (6 items: e.g., there is nothing I can do to reduce the intensity of pain). Respondents are asked to rate the extent to which each statement applies to them on a 5-point Likert scale ranging from 0 (“not at all”) to 4 (“always”). Specifically, for each statement, participants are requested to reflect on past painful experiences and indicate the degree to which they experienced these particular thoughts and feelings during pain [22]. Cronbach's alpha for the PCS in the total sample was 0.86 for the 13-item total score, 0.71 for rumination, 0.66 for magnification, and 0.78 for helplessness. This measure has been translated and its psychometric properties are good [20, 21].

### 2.6. Roland and Morris Disability Questionnaire (RDQ)

The RDQ is a 24-item checklist designed to assess pain-related disability. Patients are asked to indicate to what extent each of the statements applied to them in the last 24 hours. The RDQ score ranges from 0 (no disability) to 24 (maximum disability). In the current study, a modified version of the RDQ was used for a heterogeneous group of CPPs. Specifically, the original wording “my back pain” was changed to “my pain”; this modified version has shown excellent validity within clinical samples of patients experiencing multiple types of pain [23]. Cronbach's alpha for the RDQ in the present sample was 0.84. This measure has been translated and its psychometric properties are good [20, 21].

### 2.7. Depression Scale

Patients' depressive symptoms were assessed with the 14-item depression subscale of the Depression Anxiety Stress Scales (DASS [24]). Participants are asked to indicate the extent to which they experienced each item over the past week on a 4-point scale (0 = did not apply to me at all; 3 = applied to me very much or most of the time). Cronbach's alpha for the depression subscale, as reported by P. F. Lovibond and S. H. Lovibond [25], was excellent (*α* = 0.91). In the present sample, Cronbach's alpha for the depression subscale was 0.94. This measure has been translated and its psychometric properties are good [20, 21].

### 2.8. Family Assessment Device (FAD)

Family function was assessed using the 60-item Family Assessment Device (FAD). The FAD is based on the McMaster Model of Family Function and consists of 6 subscales [26]. In the current study, there are 3 subscales of the FAD, that is, “roles (11 items),” “communication (9 items),” and “problem-solving (6 items),” which were hypothesized to be correlated with the experience of pain based upon theoretical arguments. The FAD is scored by summing the endorsed responses (1–4) for each subscale (negatively worded items are reversed) and dividing them by the number of items in each scale. Accordingly, individual scale scores range from 1 (best functioning) to 4 (worse functioning). The FAD has been found to have high levels of internal consistency across a variety of different types of families [27] and acceptable levels of test-retest reliability [28]. In the current study, Cronbach's alpha was 0.92 for the FAD total score, 0.70 for “problem-solving,” 0.72 for “communication,” and 0.71 for “roles.”

## 3. Results

### 3.1. Participant Characteristics

Participants were 142 married patients with chronic musculoskeletal pain (96 women [67.6%], 46 men [46.4%]) lasting at least a minimum of three months. Most participants reported multiple pain locations (or diffuse pain) (48.6%) followed by pain in the back (14.8%), knee (14.8%), and feet (12%). The average duration of pain in the current sample was 46.33 months (SD = 65.69), and 74.8% of the participants were taking analgesic medication. The mean age of the sample was 45.9 years (SD = 11.9). More than one-third of the sample (35.3%; *N* = 50) had a university degree, 45.8% (*N* = 65) had at least 11 years of education, and 19% (*N* = 27) had a high school diploma.

### 3.2. Data Analysis

To evaluate the hypothetical model presented in the study, structural equation modeling (SEM) method was performed by using AMOS 20.0 [29]. SEM provides fit indices to examine the proposed relationships among variables in a model [30] and allows the relationship between multiple dependent or outcome variables to be examined simultaneously. The maximum likelihood was used to assess model fit. In line with recommendations of Byrne [31], several fit indices were used for parameter estimation. In the present study, the model fit is assessed using the following goodness of fit indices: *χ* ^2^ which is very sensitive to sample size and nonnormality of the data with a nonsignificant *χ* ^2^ implying a goodness of fit of the model to the data [32]; RMSEA [33] which is a fit measure based on population error of approximation with a RMSEA value below 0.08 indicating a close fit and values below 0.10 representing reasonable errors of approximation in the population (Table 3); moreover, CFI which is an incremental fit index [28] and represents the proportionate improvement in model fit by comparing the target model with a baseline model; normed fit index (NFI) [34]; Tucker-Lewis Index (TLI) [35]; and the Consistent Akaike Information Criterion (CAIC) [36]. For the purpose of the present study, goodness of fit was evaluated using the following statistics: NFI > 0.90, CFI > 0.90, normal chi-square (3 < *χ* ^2^/df < 2), and RMSEA and its 90% confidence interval (<0.08) [37].

### 3.3. Preliminary Analyses

The data was inspected for skewness and kurtosis. All variables were normally distributed and did not violate the underlying assumptions for the analysis (Table 1). The correlations between measures in the model (Table 2) were examined. Variance inflation factors (VIFs) were tested to check the statistical multicollinearity. All VIFs were found less than 2, which is lower than what is considered as evidence of multicollinearity (rules of thumb less than 5) [38].

### 3.4. Model Testing

**Figure 1:**
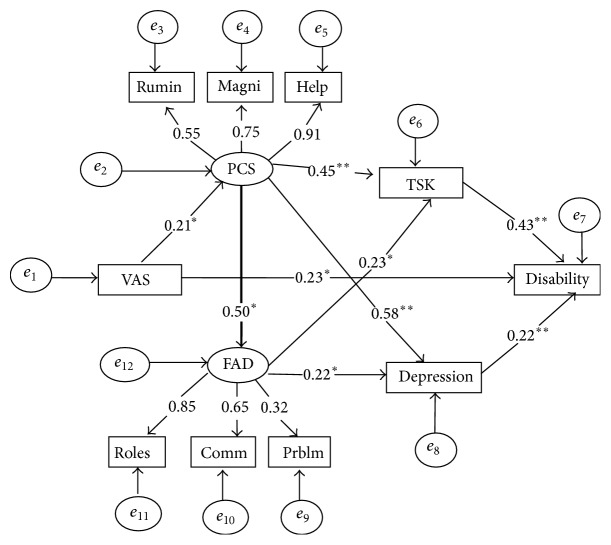
Model 1 with resulting standardized regression weights. All coefficients are significant (*p* < 0.05^*∗*^, *p* < 0.001^*∗∗*^). VAS, visual analogue scale; PCS, Pain Catastrophizing Scale; Rumin, PCS-rumination; Magni, PCS-magnification; Help, PCS-helplessness; TSK, Tampa Scale of Kinesiophobia; FAD, Family Assessment Device; Comm, FAD-communication; Prblm, FAD-problem-solving; Roles, FAD roles subscale.

The initial model is depicted in Figure 1. Pain catastrophizing, fear of movement, family function, and depression are considered as possible pathways which may lead to disability. This model assumes that pain intensity predicts disability. It also tests core aspect of the fear-avoidance model of pain in which pain catastrophizing and fear of movement mediate the relationship between pain and disability [1]. It also proposes that the relationship between pain and disability is mediated by family dysfunction and depression. In other words, this model assumes that pain catastrophizing indirectly predicts disability through family dysfunction [39] and depression [40]. It also suggests that pain catastrophizing directly predicts depression [41, 42].

The goodness of fit statistics of this model indicated an acceptable fit (*χ* ^2^ = 45.54 (30) = 1.52, *p* < 0.05, NFI = 0.90, CAIC = 116.53, TLI = 0.94, CFI = 0.96, and RMSEA = 0.06).

**Figure 2:**
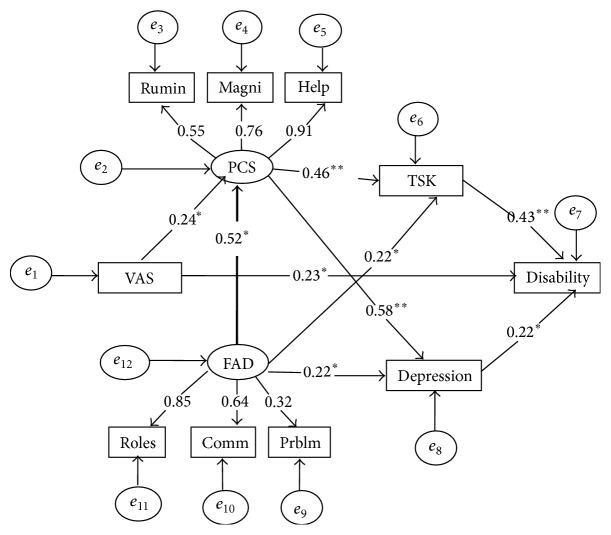
Model 2 with resulting standardized regression weights. All coefficients are significant (*p* < 0.05^*∗*^, *p* < 0.001^*∗∗*^). VAS, visual analogue scale; PCS, Pain Catastrophizing Scale; Rumin, PCS-rumination; Magni, PCS-magnification; Help, PCS-helplessness; TSK, Tampa Scale of Kinesiophobia; FAD, Family Assessment Device; Comm, FAD-communication; Prblm, FAD-problem-solving; Roles, FAD roles subscale.

**Figure 3:**
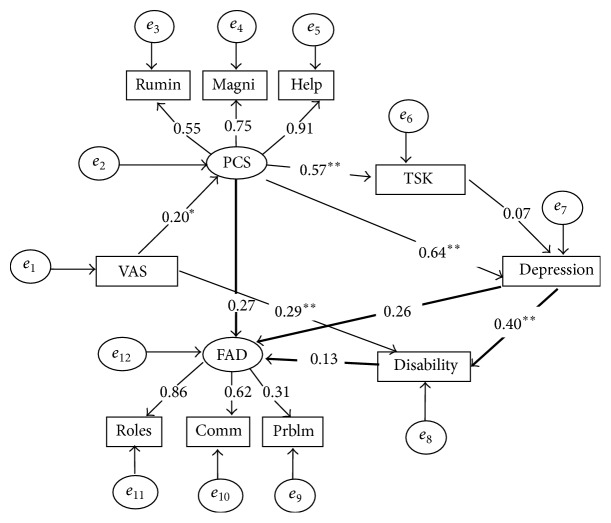
Model 3 with resulting standardized regression weights (*p* < 0.05^*∗*^, *p* < 0.001^*∗∗*^). VAS, visual analogue scale; PCS, Pain Catastrophizing Scale; Rumin, PCS-rumination; Magni, PCS-magnification; Help, PCS-helplessness; TSK, Tampa Scale of Kinesiophobia; FAD, Family Assessment Device; Comm, FAD-communication; Prblm, FAD-problem-solving; Roles, FAD roles subscale.

We also tested an alternative model which considers family function as triggering/preceding catastrophic thinking (Figure 2). The model fitted the data from acceptable to excellent fit indices (*χ* ^2^ = 43.39 (30) = 1.45, *p* < 0.05, NFI = 0.91, CAIC = 113.39, TLI = 0.95, CFI = 0.97, and RMSEA = 0.05). Overall, fit indices of the second model were superior. The standardized indirect effect of family function on depression was 0.30. The standardized indirect effect of pain intensity on depression was 0.14. The standardized indirect effect of pain intensity on disability was 0.08, while the standardized indirect effect of family function on disability was 0.31, and the standardized indirect effect of pain catastrophizing on disability was 0.32. According to this model (Figure 2), four mediators and one exogenous variable (VAS) with one endogenous variable (disability) accounted for 40% of the variance in disability, 51% of depression, 32% of fear of movement, and 32% of catastrophizing. A second alternative model (Figure 3) was tested to examine if family dysfunction may be better conceived as a consequence of depression and disability. This model was considered based on an alternative explanation that disability may contribute to family dysfunction. The goodness of fit indices for this model were unacceptable (*χ* ^2^ = 79.99 (30) = 2.67, *p* < 0.05, NFI = 0.83, CAIC = 149.99, TLI = 0.83, CFI = 0.89, and RMSEA = 0.11).

To reevaluate the final model, bootstrapping method with 1000-sample generation and 95% interval confidence was conducted to correct possible biases. The results did not change and no further finding is reported.

## 4. Discussion

In the current study, we examined the relationship between a number of individual and family-related variables to better understand CP and associated disability. Specifically, we examined the relationship between patients' catastrophizing, pain-related fear, depressive symptoms, and disability and the role of family functioning (as indexed by roles, communication, and problem-solving). Three different models were examined. Within the first model, we examined the impact of family function in the development of disability due to an increase in pain-related catastrophizing, fear of movement, and depression. The fit indices for this model were satisfactory suggesting a mediating role of family function in the relationship between catastrophizing and the outcomes of interest. However, our second model, in which family function was considered as a precursor of catastrophic thinking, revealed a more acceptable fit compared to the original model. Our third model, in which we considered family function as a consequence of depression and disability, showed unacceptable fit. Taken together, our results indicated that pain intensity is related to increased pain catastrophizing, which, in turn, contributes to the development of fear of movement and finally results in more disability. This finding fits within the fear-avoidance model of pain and is parallel with previous research [1, 43–46]. However, our findings indicate the importance of including family functioning in understanding these relationships and suggest a particularly important role of family dysfunction in understanding pain catastrophizing thoughts.

For all models tested, all paths from pain intensity to pain catastrophizing, from family function to pain catastrophizing, from family function to depression, and from depression to disability were statistically significant. As such, our results converge with previous research suggesting that pain intensity and catastrophizing are related to each other [47]. Interestingly, however, family dysfunction was a strong predictor of pain catastrophizing. This finding is consistent with previous research suggesting that passive coping strategies are related to poor family roles, communication, and problem-solving [39]. In addition, findings show that CP can impact various facets of individual and family function [3, 15, 48]. Since CP mainly restricts daily activities, personal roles of patients may be affected which in turn may influence interpersonal communication and problem-solving in family [14, 39]. The results of the current study shed more light on the notion that family dysfunction, especially in the domains of roles, communication, and problem-solving, is likely to initiate negative cognitions relating to pain (i.e., pain catastrophizing) and result in a vicious circle towards further disability through augmenting fear and depression. This finding suggests that in case of family dysfunction, indexed by hampered family roles, less effective communication, and difficulties in daily problem-solving, patients may become more likely to negatively concentrate on the pain problem and, hence, catastrophize about their pain.

Drawing upon the literature, it is likely that when patients are not able to perform their roles, other members of the family may take over their responsibilities [49–51]. Such changes in family roles can increase the unpleasant sense of inadequacy and perception of oneself of being a burden for others. Such self-perceptions are found to be related to depressive symptoms [52], which is another pathway towards more disability. Moreover, when pain extends over a long period of time, family members may respond more negatively to patient's pain behaviors which increases the likelihood of personal conflict and dysfunctional communication among family members [16, 47].

Findings further indicated that pain catastrophizing was a strong contributor of depression. This finding is consistent across studies [17, 41, 42, 53] and signifies that the more negatively the patients focus on their pain, the more the depressive symptoms they may experience are. Long-term pain impacts family life and CPPs would find it more difficult to express their needs and feelings clearly, which, in turn, contributes to the development of further maladaptive communication [54]. This study suggests that poor family function is associated with depressive symptoms in pain patients [10, 55]; moreover, family dysfunction has a significant but indirect association with patients' disability through depression and fear of movement. In fact, CPPs with higher levels of catastrophizing may develop depression when confronting family issues.

The most influential model on chronic pain, that is, the fear-avoidance model, emphasizes the role of fear of pain in the development of chronic pain [2]. The fear-avoidance model predicts that pain-related fear may lead to the development of maladaptive avoidance behavior which does not allow the person to reconsider his/her earlier beliefs about the threatening value of pain. This will further lead to disuse and can contribute to the development disability and depression [2]. Earlier studies investigated the role of cognitive family-related factors in chronic pain and suggested that biased processing of pain in partners/family members can contribute to the persistency of maladaptive beliefs in patients and further problems in the future [21, 56].

Our findings suggest that family dysfunction is another important variable, as it may also contribute to disability through its effect on catastrophizing and associated fear of pain; that is, findings indicated that family dysfunction predicts pain catastrophizing, which, in turn, predicts more fear of movement and consequent disability.

The present study findings also indicated that pain intensity moderately contributed to disability which is parallel with previous studies [4]. However, cognitive, affective, and family factors are significantly involved in explaining the disability. Finally, we found that, in addition to pain intensity, fear of movement, and depression, family function may contribute to explaining a significant proportion of the variance of disability. Therefore, pain-related disability might be better conceived of both individual and familial variables.

The present study has a number of limitations that should be considered when interpreting the results. First, because of the cross-sectional design of the study, path directions are theoretical and causality cannot be inferred. Studies employing a longitudinal design are needed to further examine the idea that chronic pain influences family functioning, which, in turn, contributes to the emergence and/or maintenance of disability. The current study is also limited by its sole reliance on single source (i.e., patient) self-report measures. Future studies will benefit from adopting a multi-informant approach by including reports of both patients and their spouses; therefore, dyadic data analysis can be used. Observational measures on family function may also complement self-report measures and provide a more fine-grained understanding of interpersonal dynamics in the context of pain. Further, measures may share substantial variance due to item content similarities. This may lead to overestimation of correlations between variables, although we checked statistical colinearity.

Despite these limitations, this study had a number of strengths. Specifically, well-validated measures were employed and SEM was used to examine relationships among variables, which has advantages over regression analysis. Further, this is one of few studies which incorporates both individual and family correlates of disability. Introducing family function enriches theoretical models of pain and disability. Our results provide additional support for the notion that catastrophizing exerts its negative effects through several pathways. Perhaps the most significant strength of the present study is to provide additional support for a hypothetical model that integrates cognitive, affective, and family factors to predict patients' disability. The suggested model is a preliminary attempt to incorporate family-related factors into pain, and obviously further research will enrich it, especially through longitudinal designs. In addition, these findings have clinical implications to develop more effective pain management programs when contextual variables are considered [21].

## Figures and Tables

**Table 1:** Psychometric properties of measures used in the study.

Variable	Indicators	N. item	*α*	M	SD
FAD	Problem-solving	6	0.70	1.87	0.37
	Communication	9	0.72	2.36	0.62
	Roles	11	0.71	2.35	0.52

PCS	Rumination	4	0.71	12.35	3.12
	Magnification	3	0.66	4.96	3.06
	Helplessness	6	0.78	10.56	5.28

TSK		17	0.80	20.51	6.02

RDQ		24	0.84	11.11	5.17

VAS		1		54.9	23.44

Dep		14	0.94	12.32	9.84

*Note*. FAD, Family Assessment Device; PCS, Pain Catastrophizing Scale; TSK, Tampa Scale of Kinesiophobia; RDQ, Roland and Morris Disability Questionnaire; VAS, visual analogue scale; Dep, depression subscale of DASS; N. item, number of items.

**Table 2:** Intercorrelation between measures.

Scale	VAS	PCS	TSK	RDQ	FAD	Dep
VAS	—	0.24^*∗*^	0.11	0.32^*∗∗*^	0.10	0.14
PCS		—	0.53^*∗∗*^	0.44	0.50^*∗∗*^	0.69^*∗∗*^
TSK			—	0.54^*∗∗*^	0.44^*∗*^	0.40
RDQ				—	0.33	0.43^*∗∗*^
FAD					—	0.52^*∗*^
Dep						—

*Note*. VAS, visual analogue scale; TSK, Tampa Scale of Kinesiophobia; PCS, Pain Catastrophizing Scale; RDQ, Roland and Morris Disability Questionnaire; FAD, Family Assessment Device; Dep, depression subscale of DASS. ^*∗*^ *p* < 0.05. ^*∗∗*^ *p* < 0.01.

**Table 3:** Goodness of fit indices.

Model	NFI	CAIC	RMSEA	IFI	CFI	TLI	*χ* ^2^	df	*χ* ^2^/df	Δ*χ* ^2^
M1	0.90	116.53	0.06	0.96	0.96	0.94	45.54	30	1.52	2.15
M2	0.91	113.39	0.05	0.97	0.97	0.95	43.39	30	1.45	36.6
M3	0.83	149.99	0.11	0.87	0.89	0.83	79.99	30	2.67	79.99

*Note*. Δ*χ* ^2^, difference between three competitive models; NFI, normed fit index; CAIC, calculated Consistent Akaike Information Criterion; RMSEA, root-mean-square-error of approximation; IFI, incremental fit index; CFI, comparative fit index; TLI, Tucker-Lewis Index.

## Additional Points

The current study investigated the role of individual and family factors in understanding chronic pain patients' disability. Three models of disability including both individual and family-related variables as contributors to chronic pain disability were examined. Findings revealed superior fit of the model that posited that family dysfunction contributes to chronic pain patients' disability through augmentation of patient catastrophic thinking, fear of movement, and depression. The current findings provide a further glimpse of the important role of family functioning in understanding chronic pain problems. Future studies employing longitudinal designs are warranted.
