# Corrigendum to “Incorporating Family Function into Chronic Pain Disability: The Role of Catastrophizing”

**DOI:** 10.1155/2017/3808520

**Published:** 2017-10-25

**Authors:** Fatemeh Akbari, Mohsen Dehghani, Ali Khatibi, Tine Vervoort

**Affiliations:** ^1^Family Research Institute, Shahid Beheshti University, G.C., Tehran 1983963113, Iran; ^2^Department of Psychology, Shahid Beheshti University, G.C., Tehran 1983963113, Iran; ^3^Psychology Department, Bilkent University, 06800 Ankara, Turkey; ^4^Interdisciplinary Program in Neuroscience, Bilkent University, 06800 Ankara, Turkey; ^5^Department of Experimental-Clinical and Health Psychology, Ghent University, 9000 Ghent, Belgium

In the article titled “Incorporating Family Function into Chronic Pain Disability: The Role of Catastrophizing” [1], an acknowledgment should be added as follows.

The authors would like to acknowledge Dr. Majtaba Habibi, a co-adviser of Dr. Akbari's thesis, for his role in the project and revising an earlier version of the manuscript.

This follows a decision by Shahid Beheshti University about an authorship claim by Dr. Habibi.
